# Clinical characteristics of patients with prenatal hydronephrosis in early postnatal period: a single center retrospective study

**DOI:** 10.1186/s12887-023-04063-5

**Published:** 2023-05-12

**Authors:** Song-Lei Gu, Xiao-Qing Yang, Yi-Hui Zhai, Wen-Li Xu, Wen-Xing Guo, Tong Shen

**Affiliations:** 1 Department of Pediatrics, Women and Children’s Hospital of Xiamen University, School of Medicine, Xiamen University, Zhenhai road 10, Xiamen, 361003 China; 2 Department of Nephrology, Children’s Hospital of Fudan University, Wanyuan road 399, Shanghai, 201102 China

**Keywords:** Hydronephrosis, Malformation, Urinary tract dilation, Urinary tract infection, Surgery

## Abstract

**Background:**

The study aims to investigate the clinical characteristics of early postnatal period in children with prenatal hydronephrosis (HN) in our single center for 8 years.

**Study design:**

The clinical data of 1137 children with prenatal HN from 2012 to 2020 were retrospectively analyzed in our center. Variables of our study mainly included different malformations and urinary tract dilation (UTD) classification, and main outcomes were recurrent hospitalization, urinary tract infection (UTI), jaundice, and surgery.

**Results:**

Among the 1137 children with prenatal HN in our center, 188 cases (16.5%) were followed-up in early postnatal period, and 110 cases (58.5%) were found malformations. The incidence of recurrent hospitalization (29.8%) and UTI (72.5%) were higher in malformation, but the incidence of jaundice (46.2%) was higher in non-malformation(*P* < 0.001). Furthermore, UTI and jaundice were higher in vesicoureteral reflux (VUR) than those in uretero-pelvic junction obstruction (UPJO) (*P* < 0.05). Meanwhile, Children with UTD P2 and UTD P3 were prone to recurrent UTI, but UTD P0 was prone to jaundice (*P* < 0.001). In addition, 30 cases (16.0%) of surgery were all with malformations, and the surgical rates of UTD P2 and UTD P3 were higher than those of UTD P0 and UTD P1 (*P* < 0.001). Lastly, we concluded that the first follow-up should be less than 7 days, the first assessment should be 2 months, and the follow up should be at least once every 3 months.

**Conclusion:**

Children with prenatal HN have been found many malformations in early postnatal period, and with high-grade UTD were more prone to recurrent UTI, even to surgery. So, prenatal HN with malformations and high-grade UTD should be followed up in early postnatal period regularly.

**Supplementary Information:**

The online version contains supplementary material available at 10.1186/s12887-023-04063-5.

## Background

Congenital abnormalities of kidney and urinary tract (CAKUT) is the most common cause of chronic kidney disease (CKD), which frequently was found in prenatal HN. Most children with HN could resolve spontaneously, while a small number of children could be progress to CKD requiring renal replacement therapy [1–2]. Therefore, precise prevention and treatment of children with HN could reduce the incidence of CKD. Recently report showed that the main causes of HN were UPJO, VUR, vesicoureteral junction (VUJO), duplication kidney (DK), posterior urethral valve (PUV), and other malformations. Among them, UPJO was the most common, followed by VUR [3]. Understanding the clinical characteristics of malformation in early postnatal period with prenatal HN after birth is crucial to precise prevention and treatment of HN.

UPJO generally occurs unilaterally in most cases, even in those with severe HN. A conservative approach seemed reasonable, but it should be frequently exercised by B-ultrasound (US) changes [4]. If there is aggravation of HN or decline in renal function, early surgical intervention was recommended for the case [3, 4]. Early surgical treatment is importance to prevent CKD, especially endoscopic treatment with insertion of a double J prosthesis [5]. Recently study showed that increasing renal pelvis collagen ratio may be clue for the early loss of renal function in congenital HN [6]. VUR refers to the reflux of urine from the bladder to ureter or renal pelvis, prone to recurrent UTI, leading to renal scarring, atrophy, and renal dysfunction syndrome [7, 8]. Generally, 11–30% children with HN will develop VUR, and the rate of spontaneous remission of moderate to mild unilateral reflux is higher in boys [3, 9]. But some children with severe HN could have renal function damage, and early surgical treatment could be conducive to preserve renal function [10]. In addition, other malformations (VUJO, DK, PUV, Other malformations, and non-malformations) also have clinical characteristics in early postnatal period.

Up to now, there are few studies on what clinical characteristics of malformations in early postnatal period with prenatal HN after birth, especially chief adverse events (recurrent hospitalization, UTI, jaundice, surgery). And we also have no foundation on the time of follow-up. So, we collected the clinical data of children with prenatal HN in our center from 2012 to 2020. And we also analyzed and explored the clinical characteristics of malformations in early postnatal period with prenatal HN after birth to lay the foundation for accurate prevention and treatment.

## Methods

### Population and Study design (Fig. 1)

Following ethics board approval (application number: KY-2022-094-K01, Date: 22 September 2022), we reviewed our prospectively-collected prenatal HN data base from 2012 to 2020 in Women and Children’s care center. Pregnant women all had US examination between 28 and 36 weeks of gestation. We enrolled 1137 fetuses (excluded abortion and stillbirth) whose US showed APD ≥ 7 mm between 28 and 36 weeks of gestation [11] from our center, and 191 Infants were followed up in our department of pediatrics at our center after birth. Infants included UPJO, VUR, VUJO, DK, PUV, other abnormalities, and non-malformations with prenatal HN and were screened for an ongoing clinical trial. Exclusion criteria included abortion, stillbirth, and no complete data. We excluded 3 Infants without complete data, and finalized a total of 188 infants in the present analysis.

**Fig. 1:**
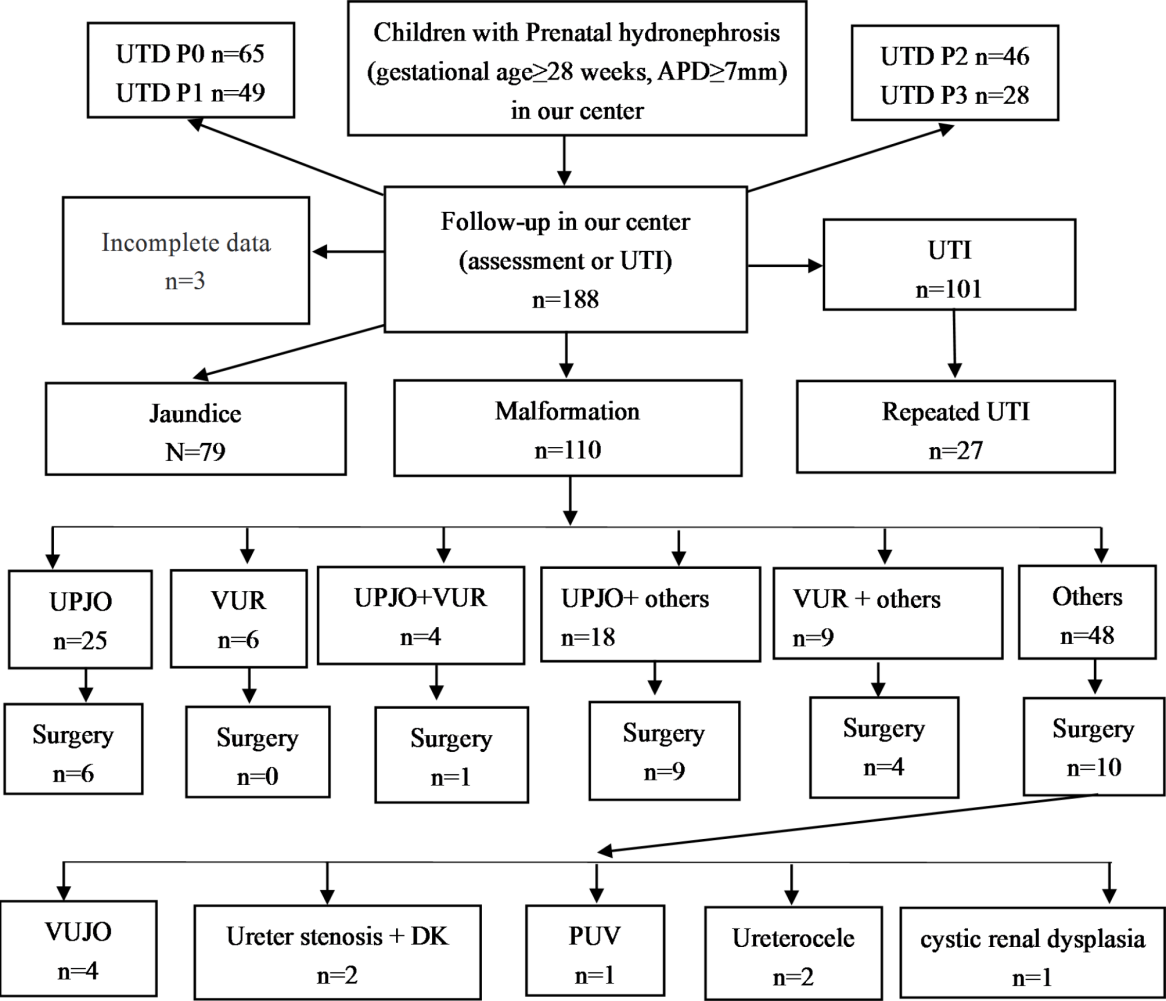
Flowchart of study population of early postnatal period in children with prenatal HN (N = 1137) followed up in our center

Notes 1: HN: Hydronephrosis, UTD: Urinary Tract Dilation, UTI: Urinary Tract Infection, UPJO: Uretero-Pelvic Junction Obstruction, VUR: Vesicoureteral Reflux, VUJO: Vesicoureteral Junction, DK: Duplication Kidney, PUV: Posterior Urethral Valve, Others: including 9 cases of VUJO, 14 cases of DK, 2 cases of PUV, and 23 cases of other malformations.

### Independent variables and outcomes of interest

We collected data on 188 infants. The groups were as follow: UPJO, VUR, UPJO combined with VUR, UPJO combined with other malformations, VUR combined with other malformations, other malformations, and non-malformations. Main concerns included the gender ratio, parity, gestational age, age of the first hospitalization, times of the repeated hospitalizations, age at the repeated hospitalizations, the interval time between hospitalizations, the jaundice ratio, the UTI ratio, UTD grade (P0, P1, P2, P3), VUR grade (I, II, III, IV, V), and the surgical intervention ratio. Furthermore, we concerned about UTI, jaundice, and surgical intervention in different UTD classifications and malformations of prenatal HN after birth.

We gathered US images for each of the enrolled infants at the mentioned above. All US results were performed and confirmed by 2 clinicians who were blinded to infant outcomes. Our primary outcomes assessed UTD grade (0, 1, 2, 3) [12], measured APD (Antero-Posterior (renal pelvis) diameter), and detected urinary tract malformations. We gathered Voiding Cystourethrogram (VCUG) images for each of the enrolled infants at the mentioned above. All VCUG results were performed and confirmed by 2 clinicians who were blinded to infant outcomes. Our primary outcomes assessed VUR grade (I, II, III, IV, V). In children presenting with dilating VUR (grade III-V), antibiotic prophylaxis (CAP) was the preferred option for initial therapy. Circumcision was considered in boys with breakthrough UTI in VUR.

The specify surgical indications in different malformations were as follow: UPJO: a dilated renal pelvis (> 30 mm) on US, or/and a significant increase of the APD on US, or/and the level of differential renal function (DRF) < 40% on renogram. VUR: febrile UTI recurrence in the context of high-grade VUR (grade III-V), or/and new renal scarring on renal static imaging, or/and having no tendency towards self-healing with severe dilating VUR (grade IV-V). VUJO: presence of persistent obstruction, or/and a significant increase of the APD on US, or/and the level of DRF decrease on renogram. PUV: endoscopic valves ablation is necessary to improves hydronephrosis and plasma creatinine when the child is medically stable, or vesicotomy can be performed in order to alleviate the obstruction when the newborn is too small (under 2Kg). DK: the case of DK with Ureter stenosis, and/or absence of function in the diseased moiety. Ureterocele: recurrent febrile UTIs, or/and deteriorating split renal function on serial renograms. Cystic renal dysplasia: solitary multilocular cystic lesions with comorbidities could not be treated, and unilateral nephrectomy was performed after excluding other unilateral renal lesions.

### Statistical analyses

Qualitative data were presented as counts and percentages and assessed using the Chi-square or Fisher-Freeman-Halton test, and Normality of data distribution were checked by Kolmogorov-Smirnov test. The correlations between two categorical variables assessed using Spearman correlation analysis. A *p* < 0.05 was considered statistically significant. All calculations were completed with commercially available statistical software (IBM® SPSS® Statistics version 22).

## Results

### Gender, parity, and gestational age in different malformations (Table 1)

Among the 1137 children with prenatal HN, 188 children were admitted to our study. Among the 188 children, 25 cases (13.3%) of UPJO, 6 cases (3.2%) of VUR, 4 cases (2.1%) of UPJO + VUR, 18 cases (11.7%) of UPJO + others, 9 cases (2.7%) of VUR + others, 48 cases (25.5%) of other malformations (including 9 cases of VUJO, 14 cases of DK, 2 cases of PUV, 23 cases of other malformations), and 78 cases (41.5%) of non-malformations.

Among the 188 children, 131 cases (69.7%) were male and 57 cases (30.3%) were female. The VUR + others (66.7%) were higher in girls compared with non-malformations, and were lower in boys (33.3%). Secondly, we found 99 cases (52.7%) of G1P1, 58 cases of G2P2 (30.9%), 8 cases of G2P1 (4.3%), 6 cases of G3P3 (3.2%), 6 cases of G3P2 (3.2%), 2 cases of G4P4 (1.1%), 4 cases of G4P2 (2.1%), 1 case of G4P1 (0.5%), and 4 cases of G5P2 (2.1%), respectively, and there was all no difference in parity (G1, ≥ G2) among all groups. In addition, we found 10 cases (5.3%) of premature, 171 cases (91.0%) of Mature, 7 cases (91.0%) of overdue fetus, respectively, and there was all no difference in parity (premature, Mature) among all groups.

### The analyses on hospitalizations of prenatal HN after birth (Table 1)

188 children were admitted to our study for assessment, the rate of hospitalization in prenatal HN after birth was about 16.5%. The analyses on age of the first hospitalization as follow: 51 (27.1%) were ≤ 7 days, 1 (0.5%) was > 7 days and ≤ 1 month, 39 (20.7%) were > 1 month and ≤ 3 months, 37 (19.7%) were > 3 months and ≤ 6 months, 24 (12.8%) were > 6 months and ≤ 1 year, 19 (10.1%) were > 1 year and ≤ 3 years, and 17 (9.0%) > 3 years, respectively. And the analyses on times of the repeated hospitalizations: 132 cases (70.2%) were once, 25 cases (13.3%) were twice, 16 cases (8.5%) were three times, 11 cases (5.9%) were four times, and 4 cases (2.1%) were five times, respectively, and there was difference on times of the repeated hospitalizations (once, ≥ twice)between malformations and non-malformations. In addition, the analyses on age at the repeated hospitalizations, 3 (1.6%) were ≤ 1 month, 13 (6.9%) were > 1 month and ≤ 3 months, 29 (15.4%) were > 3 months and ≤ 6 months, 40 (21.3%) were > 6 months and ≤ 1 year, 16 (8.5%) were > 1 year and ≤ 3 years, and 7 (3.7%) > 3years respectively. Lastly, the analyses on the interval time between hospitalizations: 10 (5.3%) were ≤ 1 month, 43 (22.9%) were > 1 month and ≤ 3 months, 17 (9.04%) were > 3 months and ≤ 6 months, 26 (13.8%) were > 6 months and ≤ 1 year, 11 (5.9%) were > 1 year and ≤ 3 years, and 1 (0.5%) > 3 years, respectively.

### UTI, Jaundice, Surgical intervention, and UTD grade in different malformations (Table 1)

Among the 188 children, 101 cases (53.7%) of UTI, 79 cases (42.0%) of jaundice, 49 cases (62.0%) jaundices were found in UTI, and 49 cases (48.5%) UTIs were complicated with jaundice. Furthermore, the incidence of UTI (72.5%) was higher in malformation, but the incidence of jaundice (46.2%) was higher in non-malformation. Meanwhile, UTI and jaundice were higher in VUR than those in UPJO, and the difference was statistically significant (*P* < 0.05). In addition, 30 cases (16.0%) children had chosen surgical intervention, and there was statistically significant difference between malformations and non-malformations. However, the rates of surgical intervention weren’t statistically significant between different malformations. Lastly, among the 188 children, 65 (34.6%) were UTD P0, 49 (26.1%) were UTD P1, 46 (24.5%) were UTD P2, and 28 (14.9%) were UTD P3, respectively. And VUR was usually lower in UTD grade (P0, ≥ P1) than UPJO + others and other malformations.

**Table 1:** Clinical characteristics of early postnatal period in children with different malformations in prenatal HN (N = 188) followed up in our center

	UPJO (N = 25)	VUR (N = 6)	UPJO + VUR (N = 4)	UPJO+ Others (N = 18)	VUR + Others (N = 9)	Others (N = 48)	No (N = 78)	*χ* ^*2*^ N = sum	*P*
Gender (male/ female)	17/8^a, b^	4/2^a, b^	3/1^a, b^	14/4^a, b^	3/6^b^	25/23^b^	65/13^a^	19.92	0.001
Gestational age (mature/ premature)	24/1^a^	6/0^a^	4/0^a^	17/1^a^	9/0^a^	46/2^a^	72/6^a^	1.45	0.975
Parity (G1/ ≥ G2)	15/10^a^	2/4^a^	2/2^a^	10/8^a^	6/3^a^	24/24^a^	40/38^a^	2.52	0.889
First hospitalization (age ≤ 6/>6 months)	16/9^a, b^	4/2^a, b^	3/1^a, b^	9/9^b^	5/4^a, b^	29/19^b^	62/16^a^	10.14	0.10
Repeated hospitalizations (age ≤ 6/>6 months)	10/5^a^ (N = 15)	4/2^a^ (N = 6)	1/2^a^ (N = 3)	9/17^a^ (N = 26)	7/7^a^ (N = 14)	11/25^a^ (N = 36)	3/5^a^ (N = 8)	8.28 N = 108	0.204
Interval time between hospitalizations (age ≤ 6/>6 months)	13/2^a^ (N = 15)	6/0^a^ (N = 6)	1/2^a^ (N = 3)	15/11^a^ (N = 26)	9/5^a^ (N = 14)	23/13^a^ (N = 36)	3/5^a^ (N = 8)	10.81 N = 108	0.076
Times of hospitalization (1/≥2)	15/10^a^	3/3^a^	2/2^a, b^	7/11^a^	4/5^a^	29/19^a^	72/6^b^	38.51	0.001
Jaundice(yes/no)	10/31^a^ (N = 41)	12/0^b^ (N = 12)	0/7^a^ (N = 7)	12/32^a^ (N = 44)	8/15^a^ (N = 23)	28/56^a^ (N = 84)	45/41^a^ (N = 86)	43.68 N = 297	0.001
UTI (yes/no)	21/20^a, b^ (N = 41)	10/2^b^ (N = 12)	7/0^b^ (N = 7)	23/21^a, b^ (N = 44)	15/8^a, b^ (N = 23)	35/49^a, b^ (N = 84)	30/56^a^ (N = 86)	24.33 N = 297	0.001
Surgical intervention (yes/no)	6/19^a^	0/6^a, b^	1/3^a^	9/9^a^	4/5^a^	10/38^a^	0/78^b^	41.13	0.001
UTD grade (P0/≥P1)	9/16^a, b^	5/1^b^	1/3^a, b^	2/16^a^	1/8^a, b^	11/37^a^	36/42^a, b^	19.85	0.002
Affected side (left/right/ Bilateral)	13/7/5^a^	1/2/3^a^	0/4/0^a^	8/6/4^a^	3/2/4^a^	21/19/8^a^	41/19/18^a^	15.74	0.158

Notes 1: Among the 1137 children with prenatal HN, 188 were followed up in our center for assessment in early postnatal period. Among the 188 children, 110 cases were found in malformation, the rate of malformation was 58.5%. Notes 2: HN: Hydronephrosis, UPJO: Uretero-Pelvic Junction Obstruction, VUR: Vesicoureteral Reflux, Others: including 9 cases of VUJO, 14 cases of DK, 2 cases of PUV, and 23 cases of other malformations, No: non-malformation, G1: Gestational 1, G2: Gestational 2, UTI: Urinary Tract Infection, UTD: Urinary Tract Dilation. Notes 3: There was statistical significance between the two groups without the same letter (a, b), and there was no statistical significance between the two groups with the same letter (a, b). *P* < 0.05 was statistically significant

### UTI, Jaundice, and Surgical intervention in different UTD grade (Table 2)

Among the 1137 children with prenatal HN, 188 cases were followed up in our center for assessment. 101 cases of UTI after birth, 27 cases repeated, and the rate of recurrent UTI was 26.7%. Furthermore, children with UTD P2 and UTD P3 were likely to recurrent UTI compared with UTD P0 and UTD P1. 79 cases of jaundice after birth, children with UTD P0 were prone to jaundice compared with UTD P1, UTD P2 and UTD P3. Meanwhile, 30 cases of surgical intervention after birth, the rate of surgical intervention was 16.0%. And the probability of surgical intervention in UTD P2 and UTD P3 were higher than those in UTD P0 and UTD P1.

**Table 2:** Clinical characteristics of early postnatal period in children with UTD grade in prenatal HN (N = 188) followed up in our center

	UTD P0 N = 65	UTD P1 N = 49	UTD P2 N = 46	UTD P3 N = 28	*χ*(2)	*P*
UTI (no/1/≥2)	26/35/4^a^	30/18/1^a^	28/8/10^b^	3/13/12^b^	47.88	< 0.001
Jaundice (no/mild/ severe)	24/40^a^ /1	32/12^b^ /5	34/11^b^ /1	19/8^b^ /1	26.57	< 0.001
Surgery (yes/no)	2/63^a^	3/46^a^	13/33^b^	12/16^b^	30.43	< 0.001

Notes 1: UTD P0: anteroposterior diameter of renal pelvis < 10 mm, UTD P1: ≥ 10 ~ 15 mm with central type Calyceal dilatation, UTDP2: > 15 mm with peripheral calyceal dilatation or urination Tube dilatation, UTD P3: ≥ 10 mm with parenchymal thinning or renal parenchyma or coustic enhancement or cortico-medullar junction (or parenchymal) cyst or bladder wall thickness Abnormal or ureteral cyst or dilated posterior urethra, UTI: Urinary Tract Infection. Notes 2: Among the 1137 children with prenatal HN, 188 were admitted to our center for assessment in early postnatal period. The rate of UTI (101) with prenatal HN was 53.7%, and 27(26.7%) UTI children repeated. The rate of jaundice (79) with prenatal HN was 42.0%. Meanwhile, 49 (48.5%) UTI children had jaundice, 49 (62.0%) jaundice children had UTI. The rate of Surgery (30) with prenatal HN was 16.0%. Notes 3: There was statistical significance between the two groups without the same letter (a, b), and there was no statistical significance between the two groups with the same letter (a, b). *P* < 0.05 was statistically significant

## Discussion

Our study found that children with HN had many malformations, and some of them were more prone to adverse events (recurrent hospitalization, UTI, jaundice, surgery) in early postnatal period. Children with HN associated malformation have frequently been in hospital, and suffered from UTI. And VUR mainly was associated with recurrent UTI. UPJO was mainly in hospital for surgery and less likely to suffer from UTI. Non-malformation or some of VUR were more prone to jaundice. Children with high-grade UTD classification of HN were more prone to recurrent UTI and surgery. But children with low-grade UTD classification were prone to jaundice. In addition, we also concluded that the first follow-up should be less than 7days, the first assessment should be 2 months, and then follow up should be at least once every 3 months.

Different malformations with HN were associated with different clinical characteristics in early postnatal period. Visuri S et al. and Zee RS et al. found that HN combined with VUR were prone to UTI, especially for 4–5 grade [13, 14]. In our study, we found that children with malformations were associated with recurrent hospitalization and UTI, and farther assumed that VUR were prone to recurrent UTI, especially for high-grade VUR(IV-V). Pogorelić Z et al. pointed out that Early surgical treatment is importance to prevent CKD for UPJO [5]. In our study, children with HN underwent surgery in almost all malformation groups in early postnatal period in the specify surgical indications. Zhang L proposed that the APD was more than 15 mm before birth, which is valuable for predicting the operation of postnatal children [15]. Rickard M et al. [16, 17] found that about 30% of children with HN need surgical treatment, and the area ratio of HN to renal parenchyma may be a good indicator to predict surgery. Recently, Melo FF et al. and Braga LH et al. found that UTD is good prediction for outcomes in HN [18, 19]. According to Zhang H et al. ’s research. HN with UTD P1 and P2 have a good prognosis and gradually reduce or disappear, while UTD P3 may further aggravate and irreversible renal function damage [20]. In our study, the probability of surgery in different malformations was the same, and UTD is good surgical prediction for all malformations. The follow-up is crucial for prevention and treatment, and the indication and time of follow-up are the crucial factors. Sarhan OM et al. proposed that prenatally detected, isolated unilateral low-grade hydronephrosis usually have a favorable prognosis, and long-term follow-up is not warranted [21]. Djahangirian O et al. proposed that an initial postnatal APD of 10 mm or greater, with a Society for Fetal Urology (SFU) grade 3–4, merits follow-up [22]. Elmaci AM et al. found that Isolated antenatal hydronephrosis with APD ≤ 20 mm would spontaneously resolve, which may be expected to happen in 3 years [23]. Green CA et al. suggested that children with prenatal HN should be evaluated once 2 months after birth, and then could be evaluated once every 3 months on average [24]. According to our research, we assumed that HN with malformations or(and) high-grade UTD (P2-P3) should be followed-up. The first time of follow-up should be less than 7 days, and the first assessment should be 2 months for prevention. Then the follow-up time should be at least once every 3 months after the first assessment.

In our study, the adverse events (recurrent hospitalization, UTI, jaundice, surgery) of HN with malformations in early postnatal period were present. However, there are also several limitations. First, 188 cases followed up in early postnatal period are the main object of our study within 1377 cases. but the other data of sample besides 188 cases is limited, which might weakly predict incidents rate of adverse event. Second, other important factors, such as renal function, physical development, and the assessment of other systems were not fully adjusted.

Our retrospective study analyzed and explored the clinical characteristics of malformation in early postnatal period with prenatal hydronephrosis after birth to lay the foundation for accurate prevention and treatment. We should pay attention to HN with VUR because of recurrent UTI, especially high-grade VUR. And CAP should be required to prevent recurrent UTI. For high-grade UTD with malformation, follow-up should be serious-minded and early surgical intervention may be required to prevent progression to CKD. So, prenatal HN with malformation should be actively follow up US in early postnatal period regular and complete imaging examination, and make preparation for possible surgical treatment for high-grade UTD.

## Conclusion

In conclusion. Children with prenatal HN have been found many malformations in early postnatal period, and with high-grade UTD were more prone to adverse events (recurrent hospitalization, recurrent UTI, jaundice, surgery). So, prenatal HN with malformations and high-grade UTD should be followed up in early postnatal period regular to lay the foundation for accurate prevention and treatment.

## Electronic supplementary material

Below is the link to the electronic supplementary material.

Supplementary Material 1

Supplementary Material 2

Supplementary Material 3

## Data Availability

Not applicable.

## Data Availability

The datasets supporting the conclusions of this article are included within the article and Supplemental section datasets. The underlying datasets are available from the corresponding author on reasonable request.

## Abbreviations

UPJO: Uretero-Pelvic Junction Obstruction
VUR: Vesicoureteral Reflux
HN: Hydronephrosis
VUJO: Vesicoureteral Junction
DK: Duplication Kidney
PUV: Posterior Urethral Valve
UTD: Urinary Tract Dilation
UTI: Urinary Tract Infection
CAKUT: Congenital Abnormalities of Kidney and Urinary Tract
CKD: Chronic Kidney Disease
APD: Antero-Posterior Diameter
VCUG: Voiding Cystourethrogram
US: Ultrasound
CAP: Antibiotic Prophylaxis
DRF: differential renal function
SFU: Society for Fetal Urology
